# Non-KAM classical chaos topology for electrons in superlattice minibands determines the inter-well quantum transition rates

**DOI:** 10.1038/s41598-024-52351-6

**Published:** 2024-03-04

**Authors:** F. Wang, M. T. Greenaway, A. G. Balanov, T. M. Fromhold

**Affiliations:** 1 School of Physics and Astronomy, University of Nottingham, Nottingham, NG7 2RD UK; 2 Department of Physics, Loughborough University, Loughborough, LE11 3TU UK

**Keywords:** Condensed-matter physics, Quantum physics

## Abstract

We investigate the quantum-classical correspondence for a particle tunnelling through a periodic superlattice structure with an applied bias voltage and an additional tilted harmonic oscillator potential. We show that the quantum mechanical tunnelling rate between neighbouring quantum wells of the superlattice is determined by the topology of the phase trajectories of the analogous classical system. This result also enables us to estimate, with high accuracy, the tunnelling rate between two spatially displaced simple harmonic oscillator states using a classical model, and thus gain new insight into this generic quantum phenomenon. This finding opens new directions for exploring and understanding the quantum-classical correspondence principle and quantum jumps between displaced harmonic oscillators, which are important in many branches of natural science.

## Introduction

Understanding the quantum-classical correspondence remains an enduring challenge of contemporary science. The transfer of concepts between classical and quantum mechanics provides insights that illuminate both. But rarely can quantitative prediction of purely quantum phenomena, such as jumps and tunnelling transitions, arise from entirely classical considerations. Conversely, it is unusual for such quantum phenomena to manifest themselves directly and quantitatively in the intricate behavior of classical systems. Here we show that quantum tunnelling transitions between the wells of a superlattice (SL) with an applied tilted simple harmonic oscillator (SHO) potential can be understood quantitatively by employing a semiclassical model^1^, thus providing a new and widely applicable link between quantum transition rates and classical dynamics.

The dynamics of particles in a superlattice with strongly coupled quantum wells can be described semiclassically using band theory. In the absence of scattering, particles that are constrained to move within a single energy band will perform Bloch oscillations under the influence of an electric field applied along the superlattice axis. If a magnetic field is then applied along the SL axis, and described by a one-dimensional SHO potential, the electron will also perform cyclotron motion perpendicular to the field direction. But if the magnetic field is tilted relative to the SL axis, it has been shown^1^ that coupling between the SHO cyclotron motion and Bloch oscillations gives rise to chaotic dynamics^2–8^. The Kolmogorov–Arnold–Moser (KAM) theorem states that for systems that are non-degenerate, which in a classical picture means that the oscillation frequency depends on the energy of the oscillator, the transition to chaos occurs gradually as the size of an applied perturbation increases. But for our system, the KAM theorem does not apply because the unperturbed system is a harmonic oscillator whose frequency is independent of energy, making it classically degenerate. Details of the KAM theorem and non-KAM chaos for degenerate systems are given in Refs.^2–8^. Non-KAM chaos occurs when a harmonic oscillator is perturbed by a plane wave and is characterised by stochastic web-like (SW) structures that abruptly appear in the particle’s phase space, along which the particle can diffuse rapidly. It has been shown to give rise to resonant peaks in the measured current-voltage characteristics of a semiconductor SL in applied magnetic and electric fields^9–11^.

A key measure of quantum transitions is the rate at which they occur, as determined by the overlap integral of the initial and final state wavefunctions. Calculation of the overlap integrals between two spatially and energetically displaced quantum simple harmonic oscillator states is a ubiquitous problem in quantum theory^12^ and relevant to many areas of natural science. They underpin the Franck–Condon principle^13,14^, which states that a quantum particle transition is most likely to occur between states whose wavefunctions have maximal spatial overlap. It is useful in many areas of physics, chemistry and biology for calculating photonic, phononic, and plasmonic transition rates between molecular energy levels^15–18^, and diverse processes including the biological mechanism behind vision^19,20^, quantum logic in molecular ions^21^, imaging molecular motion^22^ and the operation of molecular transistors^23,24^. Transitions between spatially and energetically displaced harmonic oscillators are also important for quantum optics^25,26^, understanding the Jahn-Teller effect^27–29^, quantum tunnelling between two-dimensional electron gases^30,31^, and inter-layer transport in van der Waals heterostructures^32–35^.

Here, we demonstrate that, remarkably, both the amplitude and the form of the overlap integrals between displaced harmonic oscillator states can be quantitatively determined purely by considering the phase space topology of classical stochastic webs. We find clear quantitative equivalence between the *classical* stochastic web topology and electron diffusion rates, and the *quantum* mechanical overlap integrals, which provides new insights that the tunnel coupling of adjacent SHO states corresponds to the rates of classical diffusion away from the stochastic web centre. Conversely, the tunnelling matrix elements govern the key timescales in the dynamics of a classical oscillator driven by a plane wave. This correspondence between the classical and quantum models enables us to calculate the tunnelling rate between two offset SHO states using a purely classical analysis. In the SL, on resonance, electron transport rates through the system are determined from the overlap integral between offset SHO states in adjacent quantum wells and, hence, from the associated SW topologies.

Finally, we show that SW dynamics generalise the widely-used Franck–Condon principle, which gives the transition rates between SHO states with a *specific* displacement, to the case of *arbitrarily-displaced* SHO states and classical oscillators.

## Quantum model

**Figure 1:**
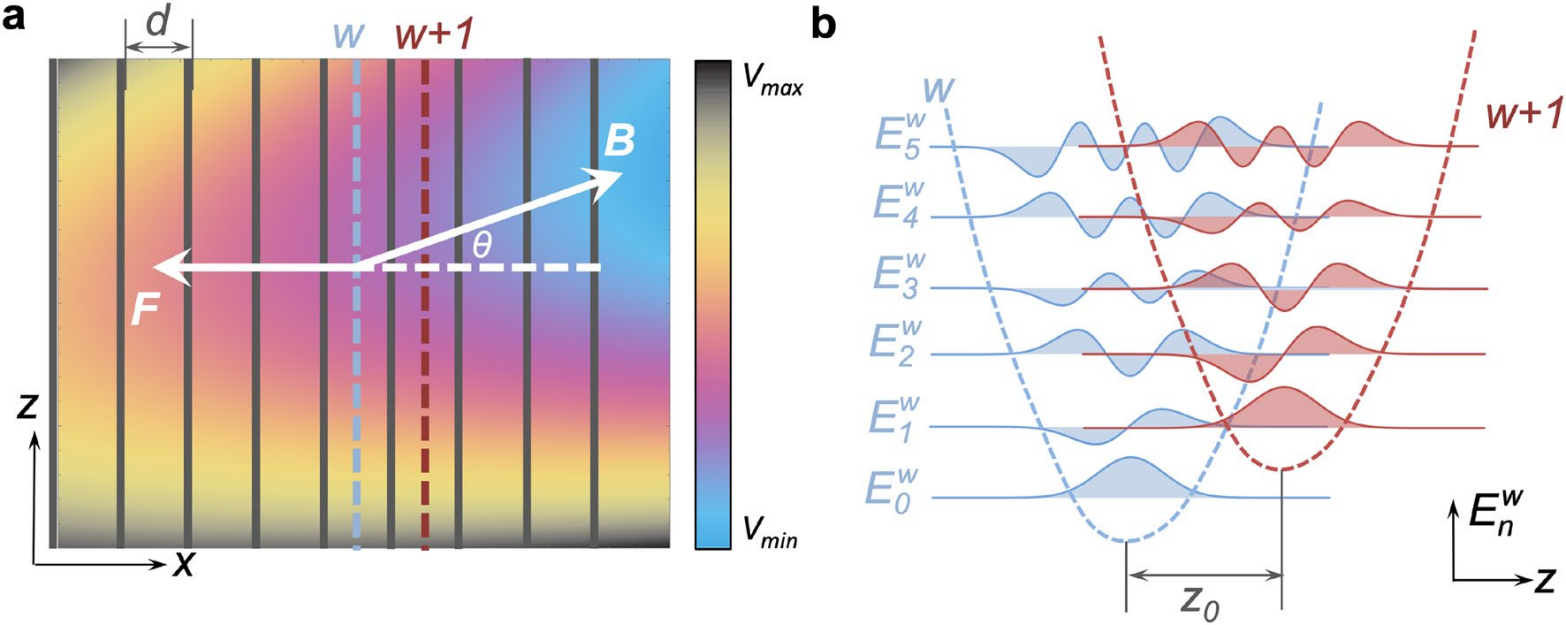
(**a**) Colour map of the total potential energy in Eq. (1) created by the constant electric field, the tilted harmonic trap, and the SL potential: vertical gray stripes indicate the tunnel barrier positions. (**b**) Wavefunction plots calculated for spatially-displaced SHO states in adjacent quantum wells with indices *w* (blue shaded curves) and $w+1$ (red shaded curves) when $r=1$. $E_n^w$ ($n=0\dots 5$) are energy levels in well *w*.

We consider electron transport through a GaAs/AlGaAs semiconductor SL with a magnetic field *B* applied at an angle $\theta$ to the superlattice axis and an electric field *F*, applied anti-parallel to the *x* axis, as shown in Fig. 1a. The Hamiltonian of this system for an electron with effective mass, $m^*$ is given by

1 $$\begin{aligned} \hat{H} = - \frac{{{\hbar ^2}}}{{2{m^*}}}\left( {\frac{{{\partial ^2}}}{{\partial {x^2}}}} + {\frac{{{\partial ^2}}}{{\partial {z^2}}}} \right) + {V_{p}}(x) + V_H(x,z) - eFx, \end{aligned}$$

here $V_p$ is the superlattice potential with period *d*, *e* is electron charge magnitude, and

2 $$\begin{aligned} V_H=\frac{m^{*}\omega _z ^2}{2} (x\sin \theta -z\cos \theta ) ^2 \end{aligned}$$

is the tilted harmonic potential that is generated by the applied magnetic field, where $\omega _z=eB/m^*\cos \theta$ is the harmonic oscillator frequency corresponding to the magnetic field component along the *z*-axis. The parameters used in our calculations are given in Section 1 of Supplementary Information (SI).

We solve the two-dimensional time-independent Schrödinger equation with the Hamiltonian in Eq. (1)^36^ to calculate the energy eigenfunctions, $\Psi$, see Eq. (7) in the SI Section 1. For small $\theta$ and *F*, the form of $\Psi$ along *z* at the centre of the quantum wells, can be accurately approximated by a series of Landau-like SHO states (arising from the SHO basis), and thus transport through the lattice can be understood in terms of tunnelling transitions between these states in adjacent wells, see Fig. 1b. When $\theta =0$, the SHO states localised in adjacent quantum wells are orthonormal and have energy $E_n^w=\hbar \omega _z(n+1/2)-weFd$ for a given energy level index, $n=0,1,2,...$, and well index, $w=0,1,2...$.

When $\theta \ne 0$ the states in adjacent wells are spatially offset by $z_0=d\tan \theta$, which breaks orthogonality and enables the electron to tunnel between multiple states in adjacent wells. In a sequential tunnelling model, the electron can transition between different wells with energy conservation when its energy, $E^w_n$ in well *w* is equal to an energy level with index $n+r$ in an adjacent well $w+1$ so that $E^w_n=E^{w+1}_{n+r}$. This resonance condition is met when the potential energy difference between the adjacent wells is $r\hbar \omega _z$, i.e. when the electric field $F=r\hbar \omega _z / ed$ and

3 $$\begin{aligned} r=\frac{eFd}{\hbar } \frac{1}{\omega _z}. \end{aligned}$$

**Figure 2:**
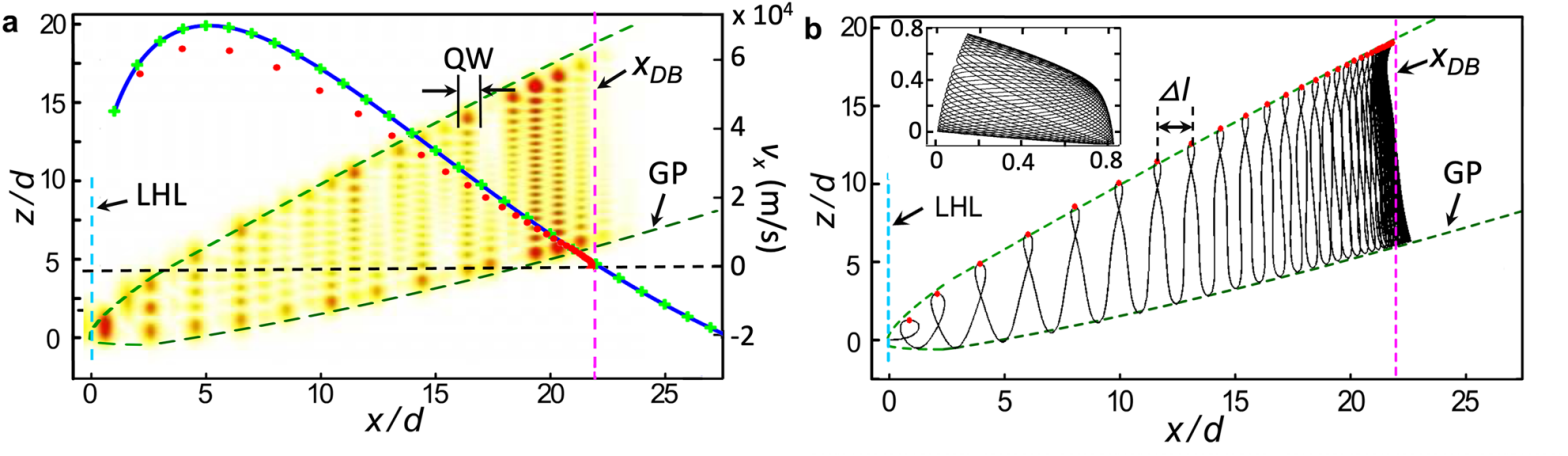
(**a**) Colour map of the electron probability density $|\Psi |^2$ (red is high), calculated when $\theta =30^\circ$, $r=1$ and $B=11$ T, with the width and location of an arbitrary quantum well (labelled QW) indicated by solid black lines. The dashed blue and magenta vertical lines show, respectively, the positions of the Left Hand Limit (LHL) and the Dynamical Barrier (DB). Dashed locus labelled GP shows the form of the Gutter Potential arising from the tilted SHO potential. Blue curve shows the function $v_0 \Xi _{n,n+1}$ and green crosses show the function $v_0 I_{n,n+1}$; red dots show $v_x(x)$ (units on right axis); see text for details. (**b**), Corresponding classical trajectory with the same parameters defined in (**a**). Red dots show the positions of the local orbital extrema along the *z* axis from which the loop spacings, $\Delta l$, are determined as shown; Inset: off resonance semiclassical orbit when $r=(1+\sqrt{5})/2$.

Our full quantum calculations show that when $r=1$, $|\Psi |^2$ extends over many lattice periods due to the resonant coupling of states with the same energy in adjacent wells, see Fig. 2a.

The calculated $|\Psi |^2$ profile reveals a regular modulation along the *x* axis with minima corresponding to the barrier regions (arbitrary well indices are labelled *w* and $w+1$ in Fig. 1), reflecting the form of the Wannier basis (see SI Section 2). In the *z* direction there are antinodes corresponding to the SHO basis. The number of antinodes increases linearly with *w*, i.e. the probability density in well $w = 0$ at has a single antinode corresponding to the $n=0$ SHO state, in well $w = 1$ there are two antinodes corresponding to the $n=1$ SHO state, and so on.

We find that $|\Psi |^2$ is fully bounded in the $(x-z)$ plane; a phenomenon which arises from three different mechanisms. The left-hand limit of $|\Psi |^2$ (labeled LHL in Fig. 2) is due to total energy conservation. The top and bottom barriers are created by the tilted SHO “gutter” potential (labeled GP). There is an also effective right-hand barrier that limits the extent of $|\Psi |^2$ along the *x*-axis (dashed vertical line labelled DB). To investigate this effective barrier further, we now consider the transition rates between adjacent wells.

Fermi’s Golden rule states that the transition rate between two quantum states depends on their overlap integral. When the electric field is tuned to the energy resonance condition, i.e. *r* is a positive integer, the *n*-th SHO state in well *w* is aligned with the $(n+r)$-th state in adjacent well $w+1$ and the two states are spatially displaced by $z_0$ along the *z* axis, see Fig. 1b. The overlap integral

4 $$\begin{aligned} I_{n,n+r} = \smallint {\phi _n}(z){\phi _{n+r}}(z+z_0)dz , \end{aligned}$$

where ${\phi _n} = {c_n}{h_n}(z/l_B)\exp ( - {z^2}/2l_B^2)$ is the $n^{th}$ SHO state, $c_n=(2^n n!)^{-1/2}(\pi l_B^2)^{-1/4}$ normalises the wavefunction, $h_n$ is the $n{th}$-order Hermite polynomial, and $l_B=\sqrt{\hbar /m \omega _z}$ is the SHO length scale along the *z* axis. The green crosses in Fig. 2a show the dependence of $I_{n,n+1}$ on the *x* co-ordinate, $x_n = (\frac{n}{r}+1)d$, of the barrier that separates wells *w* and $w + 1$, taking system parameters corresponding to the $|\Psi |^2$ plot shown in the figure. The plot reveals that $|\Psi |^2$ vanishes, i.e. is bounded by the magenta dashed line, when $I_{n,n+1}=0$. Consequently we see that the “Dynamical Barrier”, which limits the spatial extent of the eigenstate on the right-hand side, is equivalent to the point where there is a suppression of tunnel coupling between adjacent quantum wells of the superlattice. In the next section we show that this dynamical barrier, which in the quantum analysis arises from the detailed form of the offset simple harmonic oscillator wavefunctions, also manifests itself in the semiclassical dynamics of the system.

## Semiclassical model

In Refs.^1,9^, it was shown that a semiclassical model can be used accurately to model charge transport through the miniband of a SL with strongly coupled quantum wells. In this case, the dispersion relation for electrons in the first miniband is given by $E({k_x}) = \Delta _b[1-\cos (k_x d)]/2$, where $\Delta _b$ is a miniband width. On application of a tilted magnetic field, the equation of motion of the *z* component of the electron’s momentum, $p_z$ can be shown to satisfy

5 $$\begin{aligned} \ddot{p}_z + {\omega _z}^2{p_z} = - C \sin \left( \frac{z_0 p_z}{\hbar } - \omega _B t\right) . \end{aligned}$$

This equation describes a classical simple harmonic oscillator with natural frequency $\omega _z$ driven by a time (*t*)-dependent plane wave. Here, $\omega _B=eFd/\hbar$ is the Bloch oscillation frequency due to the applied electric field and the constant $C=({\Delta _{b}/2m^{*})} (z_0/l_B^4)$ . The position vector (*x*, *y*, *z*) and the other momentum components $p_x$ and $p_y$ can be determined from $p_z$^1^. This system is degenerate (in the sense that the frequency of the SHO is independent of its energy) and is known to exhibit non-KAM chaos, characterised by the formation of intricate SW patterns in phase space, which was first discovered and studied by Zaslavsky et al.^6–8^.

When $\theta =0^{\circ }$, electron motion along the *x* direction is decoupled from motion along the *z*-axis. The electron performs Bloch oscillations along the *x* axis and simple harmonic motion (with frequency $\omega _z$) along the *z* axis. Increasing $\theta$ from $0^\circ$ couples the Bloch and simple harmonic cyclotron motion causing the electron orbits to become chaotic. For most field values the extent of the electron’s trajectory along the $x-$axis is small, see inset in Fig. 2b, and therefore the electrons have a small drift velocity. But when $\omega _B$ and $\omega _z$ are commensurate, i.e. when $r=\omega _B/\omega _z$ is an integer, the trajectories become highly extended along the *x* axis, see Fig. 2b. This classical resonant condition, is equivalent to the alignment of quantised SHO energy levels in adjacent wells, see Eq. (3). Comparison between the form of $|\Psi |^2$ in Fig. 2a and the electron orbits calculated for the same system parameters in Fig. 2b reveals a striking agreement between their bounded regions. We find that the same spatial constraints, which determine which region of space the electron can access, are imposed on both the classical trajectory and $|\Psi |^2$ by the LHL, GP and, in particular, the Dynamical Barrier.

## Comparison of classical stochastic webs & quantum wigner functions

**Figure 3:**
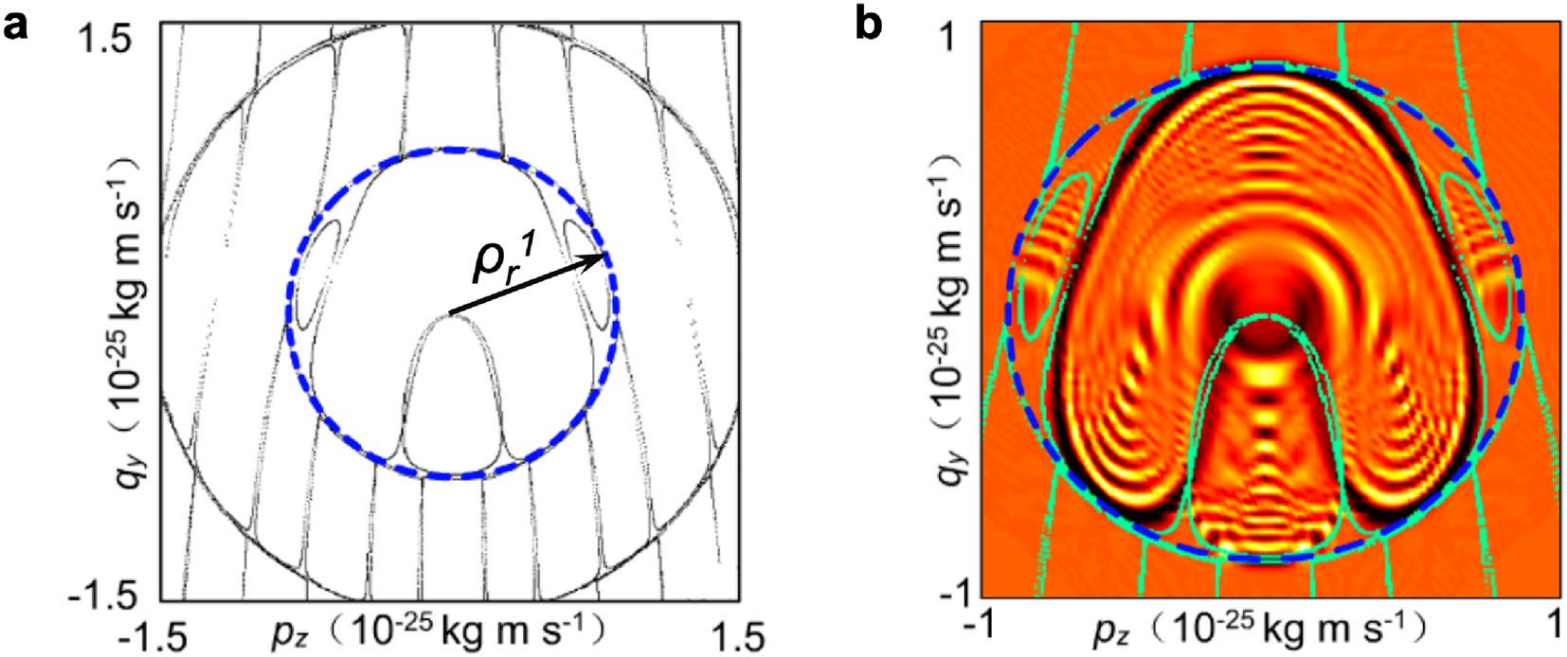
(**a**) Poincaré section calculated for $r=1$ and corresponding to the real space classical trajectory shown in Fig. 2b. (**b**) Wigner function corresponding to quantum eigenstate shown in Fig. 2a. Overlayed green dots show the classical Poincaré section shown in (**a**). Blue dashed circles in (**a**) and (**b**) show the position of the first (i.e. innermost) SW ring.

To understand the origin of the DB from a classical perspective, in Fig. 3a we show the Poincaré section $p_x=0$ of the classical particle trajectory in the $(p_z, q_y)$ phase portrait, where $q_y=\dot{p}_z/\omega _z$. We also define the polar coordinates of the particle in the phase portrait, $(\rho ,\Theta )$, where $\rho =\sqrt{q_y^2+p_z^2}$ and $\Theta$ is the angle measured with respect to the $q_y$ axis. The particle maps out an intricate SW structure, comprising an infinite set of concentric ring filaments crossed by vertical filaments that extend to infinity in each direction. The vertical filaments act as conduction channels through which the particles can diffuse rapidly, enabling the orbits, modelled by Eq. (5), to become highly spatially extended and so enhancing electrical current through the SL^9,11,37^. Even though the web filaments extend to infinity, transport through them is limited by their thickness, which depends exponentially on $\rho$^38^. Therefore, there is an exponentially small probability that the electron will continue to move out along the vertical filament towards the second ring. Instead, the particle’s trajectory becomes “trapped” on the first ring for an extended time. The radius, $\rho _r^{i}$, of the *i*th web ring for the resonance with the given integer *r* (see Fig. 3a) corresponds to the root, $\rho _r^{i} z_0$, of the Bessel function of the first kind^1^, i.e. for which

6 $$\begin{aligned} {J_r}\left( \frac{\rho _r^{i} z_0}{\hbar }\right) =0. \end{aligned}$$

It can be shown that the *x* position of an electron, starting from rest at the origin, has the following dependence on $\rho$

7 $$\begin{aligned} x(\rho ) = \frac{\rho ^2}{2m^*eF}. \end{aligned}$$

Therefore, if the electron’s orbit is constrained within the first ring of the SW in phase space, it will also have limited extent along the *x*-axis in real space, having a right-hand bound at $x(\rho _r^1)=\Delta x_r^1$. The vertical magenta dashed lines in Fig. 2a,b show the position of $\Delta x^1_r$, which coincides with where the classical trajectory and quantum wavefunction are constrained by the DB when $B = 11$ T, $\theta = 30^o$ and $r=1$.

In Fig. 3b, we show the Wigner function $W(p_z,q_y)$ corresponding to the wavefunction in Fig. 2a. The Wigner function is the quantum analogue of the semiclassical Poincaré section and is calculated by mapping $(p_z, q_y)$ onto $(x, z, p_x, p_z)$ using

8 $$\begin{aligned} \begin{aligned} W(p_z,q_y)=\frac{1}{4\pi ^2 \hbar ^2}\int _{-\infty }^{\infty }\int _{-\infty }^{\infty }\Psi ^*(x+\lambda _x,z+\lambda _z)\\ \times \Psi (x-\lambda _x,z-\lambda _z)e^{2ip_z\lambda _z/\hbar }d\lambda _xd\lambda _z. \end{aligned} \end{aligned}$$

$W(p_z,q_y)$ has a strikingly similar form to the semiclassical Poincaré section (Fig. 3a). In particular, its extent is limited by the first ring of the classical SW, shown by the dashed blue circle. This further demonstrates the equivalence between the quantum and classical pictures of the electron dynamics.

Note that when *C* in Eq. (5) is large, the channel width of SW filament is also large^8^ and the electron has a higher probability of continuing outwards along the SW filament to the second ring. However, for the parameters we consider here, this only occurs when $\theta >60^{\circ }$. Like the semiclassical trajectory, in this case the quantum energy eigenstates also extend beyond the position of the DB.

## Classical calculation of quantum tunnelling rates

In this section, we demonstrate how the SW topology determines the electron’s semiclassical velocity dependence on its position, *x*, and, by linking the quantum mechanical tunnelling rates to the local semiclasscial velocity, how the form of the SW determines the overlap integral between simple harmonic oscillator states. To investigate the connection between the position and velocity of the electron, we rewrite the semiclassical Hamiltonian in terms of the canonical action-angle variables $(\mathscr {I},\Theta )$, where

9 $$\begin{aligned} \rho ^2 \equiv \frac{2\mathscr {I}}{\omega _z } \propto x, \end{aligned}$$

$\mathscr {I}$ is action and $\Theta$ is the polar angle in the phase space $(p_z,q_y)$, see SI Section 4 for more details. In these coordinates, the electron’s velocity, $\dot{x}$, has the form^39^

10 $$\begin{aligned} \dot{x}(\rho ,t)=\dot{x}_1(\rho )+\dot{x}_2(\rho ,t), \end{aligned}$$

where $\dot{x}(\rho ,t)$ has a time-independent part, $\dot{x}_1(\rho )$, and a time-dependent part, $\dot{x}_2(\rho ,t)$, which satisfy

11 $$\begin{aligned} \dot{x}_1(\rho )&=v_0 J_r\left( z_0\rho /\hbar \right) \cos \Theta , \end{aligned}$$

12 $$\begin{aligned} \dot{x}_2(\rho ,t)&= v_0 \sum \limits _{m\ne r} J_m(z_0\rho /\hbar ) \sin \left[ \frac{m}{r}\Theta -(1-\frac{m}{r})\omega _B t\right] . \end{aligned}$$

here, $v_0= \Delta _b d/(2\hbar )$ is the maximum speed of the electron within the miniband. To find how $\dot{x}_1$ changes with *x* when an electron travels along a SW filament (i.e. when $\Theta =0$), we combine Eqs. (7) and (11) to obtain

13 $$\begin{aligned} \dot{x}_1(x) = v_0 J_r\left( \frac{z_0\sqrt{2meFx}}{\hbar }\right) . \end{aligned}$$

Quantum mechanically, the propagation of the electrons along the SL axis is associated with transitions between the states in adjacent wells. Therefore, the local electron velocity is determined by the tunnelling rate. It is reasonable to assume that the maximum electron miniband velocity, $v_0$, is realised where there is maximum overlap between the states in adjacent wells. Therefore we make the ansatz,

14 $$\begin{aligned} \dot{x}_1(x) \approx v_0 I_{n,n+1}. \end{aligned}$$

In Fig. 2a we compare the values of $\dot{x}_1(x)$ determined using (13) and (14), which are shown by the blue curve and green crosses, respectively. These values show excellent agreement, justifying the assumption in Eq. (14).

We also compare the analytically obtained $\dot{x}_1$ values with the numerically-determined electron velocity, $v_x(x)$, along the upper edge of the orbit shown in Fig. 2b. We define $v_x(x)=\Delta l(x)/\tau _z$, where $\Delta l(x)$ is the spacing between adjacent orbital peaks (see red dots in Fig. 2b), and $\tau _z=2 \pi / \omega _z$ is the oscillation period along the *z* axis. The red filled circles in Fig. 2a show the numerically calculated values of $v_x(x)$. We find good quantitative agreement between $v_x(x)$ and the analytical $\dot{x}_1(x)$ values, confirming that $\dot{x}_1$ defines the propagation of the electron along *x*. The slightly lower values of $v_x(x)$ could be explained by a contribution from the time-dependent component of electron velocity $\dot{x}_2(\rho ,t)$, which is not accounted for by Eqs. (13) and (14). This additional component could act to increase the time taken for the electron to return to the upper edge of the trajectory presented in Fig. 2b.

Comparison of Eqs. (13) and (14) suggests a general approximation for the overlap integral

15 $$\begin{aligned} I_{n,n+r} \approx J_r \left( \sqrt{2n+r+1} \frac{z_0}{l_B}\right) =\Xi _{n,n+r}. \end{aligned}$$

**Figure 4:**
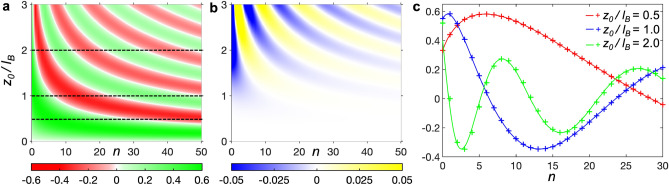
(**a**) Colour map of the calculated overlap integrals $I_{n,n+1}(n,z_0/l_B)$ (scale below). (**b**) $I_{n,n+1}-\Xi _{n,n+1}$ (scale below). (**c**) Comparison of $I_{n,n+1}$ (crosses) and $\Xi _{n,n+1}$ (lines) when $z_0/l_B=0.5, 1.0, 2.0$ (legend inset).

This approximation is also confirmed by the close similarity of the Taylor expansions of $I_{n,n+r}$ and $\Xi _{n,n+r}$ which exhibit the same asymptotic behaviour (see SI Section 5). To explore the approximation in Eq. (15) further, we investigate the dependence of the overlap integral $I_{n,n+1}$ on *n* and the ratio between the displacement of the oscillator states $z_0$ and the natural length scale of the states $l_B$, see Fig. 4a. When $z_0/l_B=0$, the harmonic oscillator states are orthogonal and uncoupled implying $I_{n,n+1}=0$ for all *n*. Increasing $z_0$ breaks orthogonality, and the overlap integral becomes finite as adjacent states couple. We find that $I_{n,n+1}$ oscillates between 0.6 and $-0.4$ with increasing $z_0$.

The accuracy of Eq. (15) is illustrated in Fig. 4b, where the dependence of $|I_{n,n+1} - \Xi _{n,n+1}|$ is shown for the same range of *n* and $x_0/l_B$. It reveals that for $z_0/l_B\lesssim 2$, $|I_{n,n+1} - \Xi _{n,n+1}|$ is close to zero reflecting the high accuracy of the approximation in Eq. (15) for this set of parameters. This correspondence is also demonstrated in Fig. 4c where the variations of $I_{n,n+1}$ and $\Xi _{n,n+1}$ with *n* are shown to be in excellent quantitative agreement for various values of $z_0/l_B$. When $z_0/l_B\gtrsim 2$ and *n* is small, $|I_{n,n+1}-\Xi _{n,n+1}|>0$ and the models diverge. Here the correspondence between the quantum and classical models breaks down due to either spatial localisation of the states (for small *n*), or, equivalently, large spatial separation of the states (large $z_0$). In SI Section 7 we provide more detail on how the length scales of the SHO states, and their dependence on *n*, determine the regimes of correspondence. We also demonstrate that expression (Eq. 15) is applicable when $r>1$.

It is important to note that expression (Eq. 15) does not depend on the parameters of the lattice, i.e. *d* and $\Delta _b$, only on the properties of the SHO states, $l_B$ and $z_0$. Therefore Eq. (15) is generally valid irrespective of the particular physical system that is described using displaced SHO states.

## Conclusion

In conclusion, we have found a remarkable link between two seemingly disparate dynamical concepts, namely the classical topology of a SW, and quantum tunnel coupling between displaced SHO wavefunctions. Using a semiconductor SL with an applied tilted magnetic field as practical example, we show that the width of the resonant delocalised classical electron trajectories and the quantum energy eigenstates can both be determined from the radii of the rings of the SW in the classical Poincaré section. Remarkably, we also find that the tunnel coupling between adjacent wells in the lattice, determined by the overlap integrals of Landau states in those wells, can also be determined purely from the topology of the SW and, more specifically, that the form of the Bessel function describes both the ring radii *and* the diffusion rate through the vertical filaments. Our analysis therefore provides a new picture of tunnelling between off-set SHO states in terms of the topology of SWs and, conversely, provides new insights into stochastic web transport in terms of quantum jumps. Our work therefore establishes correspondence between non-KAM chaos and quantum transitions between displaced harmonic oscillators, which are of fundamental importance in many areas of physics and chemistry. In particular, our work generalises the Franck–Condon principle and so provides deeper understanding of it. Whereas the Franck–Condon principle states that quantum transitions between offset SHO states are most likely when their separation is the sum of the radii of the corresponding classical orbits, our analysis shows that transitions between SHO states with *arbitrary* spatial separation can be calculated directly from classical stochastic web topology and diffusion rates. Our results provide insights into the quantum-classical correspondence in a system demonstrating non-KAM chaos. In future research it will be interesting to investigate similar relations in systems that exhibit the more commonly observed KAM chaos. Another topic of practical importance is the effect of disorder on the quantum-classical correspondence. Previously, within a semi-classical picture, we found that although low-amplitude noise has almost no effect on electron diffusion in our system, moderate noise can produce non-trivial phenomena including the enhancement electron mobility^40^. However, there has not been a comprehensive study of these phenomena from a classical perspective, and, to our knowledge, they have not been studied within the quantum realm.

### Supplementary Information

Supplementary Information.

## Data Availability

The datasets generated during and analysed during the current study are available from the corresponding author on reasonable request.
