# Supplementary material for: Non-KAM classical chaos topology for electrons in superlattice minibands determines the inter-well quantum transition rates
Source: Sci Rep. 2024 Mar 4;14:5269. doi: 10.1038/s41598-024-52351-6 (PMC10912705; doi:10.1038/s41598-024-52351-6)
Supplement: Supplementary file 1 — Supplementary Information. [file 41598_2024_52351_MOESM1_ESM.pdf]

Non-KAM classical chaos topology for electrons in superlattice minibands determines the inter-well quantum transition rates - supplementary information

F. Wang, M.T. Greenaway, A.G. Balanov, and T.M. Fromhold

1 The semiconductor superlattice

The semiconductor superlattice that we consider is a heterostructure comprising alternating layers of GaAs and AlGaAs used in previous experiments[1], see Fig S1(a). Modulation of the conduction band edge along the structure gives rise to a periodic potential, see Fig S1 (b). InAs monolayers at the centre of each GaAs/AlAs quantum well lower the energy of the first miniband, thus reducing inter-miniband Zener tunnelling and facilitating electron injection from the emitter contact. An electric field, **F**, applied antiparallel to the superlattice axis creates a linear electrostatic potential, $V_A = -eFx$, and a magnetic field, **B**, applied at an angle θ to the SL axis, induces an effective SHO potential $V_H = m^* \omega_z^2 (x \sin\theta - z\cos\theta)^2/2$ where $\omega_z = eB/m^* \cos\theta$. For the SL under consideration the effective mass $m^* = 0.069 m_e$ for electron motion in the $y-z$ plane, the band width is $\Delta_b = 19.1$ meV and the lattice period $d = 8.241$ nm.

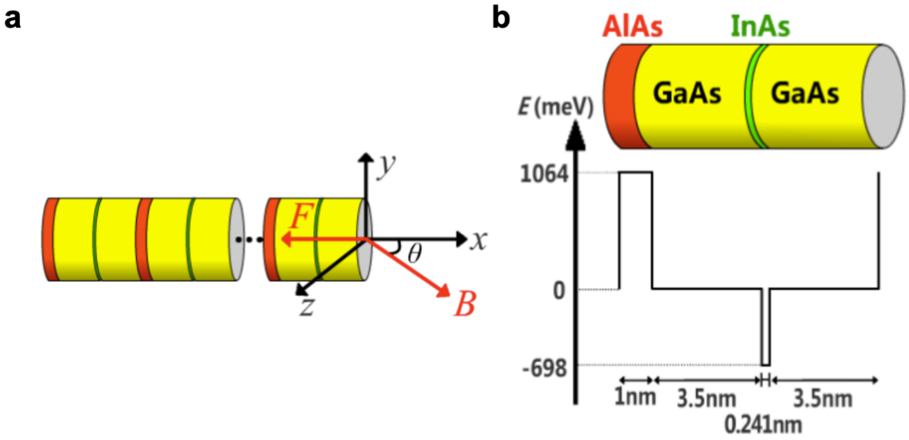

Figure S1. **(a)** Schematic diagram of the model superlattice structure used in our calculations, which consists of 61 GaAs/AlAs quantum wells. An InAs mono-layer at the centre of each quantum well lowers the energy of the first miniband, thus reducing interband tunnelling and facilitating electron injection into the SL miniband. An electric field, **F**, applied antiparallel to the superlattice axis, accelerates the electron and a magnetic field, **B**, applied at an angle θ to the superlattice axis provides a harmonic trap. **(b)** Composition of a single period of the semiconductor superlattice and (below) the corresponding electron potential energy profile produced by the conduction band offset in the different materials.

2 Calculation of quantum wavefunctions

The Hamiltonian of an electron in the superlattice can be written in the form

$$\hat{H} = \hat{H}_x + \hat{H}_z + \Delta\hat{H} = \hat{H}_0 + \Delta\hat{H}, \tag{S.1}$$

where

$$\hat{H}_x = -\frac{\hbar^2}{2m^*}\left(\frac{\partial^2}{\partial x^2}\right) + V_p(x),$$
$$\hat{H}_z = -\frac{\hbar^2}{2m^*}\left(\frac{\partial^2}{\partial z^2}\right) + \frac{1}{2}m^*\omega_z^2 z^2, \tag{S.2}$$
$$\Delta\hat{H} = -eFx + \frac{1}{2}m^*\omega_z^2 x^2 \sin^2\theta - m^*\omega_z^2 xz\sin\theta\cos\theta.$$

The solutions of the eigenvalue equation

$$\hat{H}_0\psi_g^0 = E_g\psi_g^0 \tag{S.3}$$

are used as the basis set for constructing the full Hamiltonian matrix. For $\hat{H}_x$, we expand the periodic lattice potential as a Fourier series so that Bloch states are constructed for a given wavevector k[1]. As the Bloch functions have infinite extent along the x-direction, we use the Wannier functions which are an alternative set of localised basis states[2,3] and have the form

$$\phi_{m,\alpha}(x) = N_{m,\alpha}\frac{d}{2\pi}\int_{-\pi/d}^{\pi/d} e^{-imkd}\psi_{k,\alpha}(x)dk, \tag{S.4}$$

where $N_{m,\alpha}$ is a normalisation factor, m is the index of the quantum well that the Wannier function is centred on and α is the band index.

Above, $\hat{H}_z$ is a Hamiltonian for simple harmonic oscillator, which has eigenstates

$$\varphi_n(z) = c_n h_n(z/l_B)\exp\left(-\frac{z^2}{2l_B^2}\right), \tag{S.5}$$

where $c_n = (2^n n!)^{-1/2}(\pi l_B^2)^{-1/4}$, h_n is the Hermite polynomial and $l_B = \sqrt{\hbar/m^*\omega_z}$ is the length scale of the SHO state along the z axis (see main text).

Therefore by using the basis states from Eqs. (S.4) and (S.5) we find that the final solutions to the Schödinger Equation

$$\hat{H}\psi_g = E_g\psi_g, \tag{S.6}$$

with a Hamiltonian of the form Eq. (S.1), are of the form

$$\psi_g(x,z) = \sum_{m,n,\alpha} C_{m,n,\alpha}\phi_{m,n}(x)\varphi_n(z), \tag{S.7}$$

where the $C_{m,n,\alpha}$ coefficients are found from the solution of the Hamiltonian matrix equation[1]. We diagonalize the Hamiltonian using MATLAB software to find the eigenstates. Note that in the main text, for brevity we label the energy eigenstates generically as $\Psi(=\psi_g(x,z))$ and omit reference to any specific energy eigenstate index g.

3 Calculation of Overlap Integrals

Consider the coordinate transformations $x \to (x+d)$ and $z \to (z + d\tan\theta)$, which results in a modified Hamiltonian

$$\hat{H}(x+d, z+d\tan\theta) = H(x,z) + eFd. \tag{S.8}$$

This transformation causes the electron to gain kinetic energy equal to eFd, but leaves the magnetic potential unchanged. As discussed in the main text, setting $F = r\hbar\omega_z/d$ makes the nth energy level in well w isoenergetic with the $(n+r)$th level in well $(w+1)$, and the energy eigenstates in these adjacent wells are centred on z and $z = z + d\tan\theta$ respectively. In this case, the tunnel coupling between adjacent quantum wells can be approximated by the overlap integral between the SHO states in wells w and $w+1$.

We use the following analytical equation to calculate the overlap integrals of two SHO states of order n and $n+r$ respectively which have orbit centers that are displaced by z_0[4,5].

$$\begin{aligned} I_{n,n+r} &= \int \phi_n(z)\phi_{n+r}(z-z_0)dz \\ = & \quad \exp\left(-\frac{z_0^2}{4l_B^2}\right)\cdot\sum_{j=0}^{n+r}\sum_{i=0}^{n}\frac{(-1)^i(-z_0/\sqrt{2}l_B)^{i+j}}{i!j!}\times\sqrt{\frac{(n+r)!n!}{(n+r-j)!(n-i)!}}\delta_{n+r-j,n-i} \end{aligned} \quad (S.9)$$

4 Semi-classical Hamiltonian and its form in action-angle variables

We consider the semiclassical Hamiltonian

$$H = E(p_x) + \frac{p_y^2+p_z^2}{2m^*} - eFx, \quad (S.10)$$

where $m^* = 0.069m_e$ is the electronic effective mass in the $y-z$ plane, and $E(p_x)$ is the lower non-parabolic miniband dispersion curve, which can be expanded as the following Fourier series

$$E(p_x) = a_0 - \sum_{n=1}^{\infty} a_n \cos\frac{np_xd}{\hbar}. \quad (S.11)$$

To include the effect of the magnetic field, we choose the gauge in which the magnetic vector potential

$$\mathbf{A} = [0, B(x\sin\theta - z\cos\theta), 0]. \quad (S.12)$$

Then the momentum p_y in Eq. (S.10) is replaced by

$$q_y = p_y + eA_y(x,z) = p_y + eB(x\sin\theta - z\cos\theta). \quad (S.13)$$

Thus the full semiclassical Hamiltonian, H, is

$$H = E(p_x) + \frac{1}{2}m^*\omega_c^2[x\sin\theta - (z-z_0)\cos\theta]^2 + \frac{p_z^2}{2m^*} - eFx, \quad (S.14)$$

where $\omega_c = eB/m^*$ and $z_0 = p_y/eB\cos\theta$.

For the simulation of the semi-classical dynamics, we evolved the trajectories in time using the 6th order Runge-Kutta method in C++.

To understand the form of the electron's trajectory it is useful to rewrite the semiclassical Hamiltonian in terms of action-angle $\mathscr{I}, \Theta$ variables as,

$$H(\mathscr{I},\Theta,t) = -CJ_r(K\rho)\cos\Theta - C\sum_{m\neq r} J_m(K\rho)\cos\left(\frac{m}{r}\Theta - (1-\frac{m}{r})\omega_B t\right), \quad (S.15)$$

where

$$\rho^2 \equiv \frac{2\mathscr{I}}{\omega_z} \propto x, \quad (S.16)$$

and Θ is the polar angle of the electron's position in the stochastic web measured with respect to the q_y axis and is defined using the expression $p_z = \rho\sin\Theta$.

Using these expressions with Hamilton's equations of motion we obtain the following expression for $\dot{x}$:

$$\begin{aligned} \dot{x} &= \frac{1}{\omega_z m^* eF}\dot{\mathscr{I}} && (S.17) \\ &= -\frac{1}{\omega_z m^* eF}\frac{\partial H}{\partial \phi} && (S.18) \\ &= \frac{\Delta_b d}{2\hbar}\left[\chi_1(\rho) + \chi_2(\rho,t)\right], && (S.19) \end{aligned}$$

where

$$\begin{aligned} \chi_1(\rho) &= J_r(K\rho)\cos\phi && (S.20) \\ \chi_2(\rho,t) &= \sum_{m\neq r} J_r(K\rho)\sin\left(\frac{m}{r}\phi - (1-\frac{m}{r})\omega_B t\right). && (S.21) \end{aligned}$$

5 Comparison of $I_{n,n+r}$ and J_r

As described in the Section "Classical calculation of quantum tunnelling rates" of the main text, for a large region of the parameter space when $z_0/l_B \leq 2$,

$$I_{n,n+r} = J_r\left(\sqrt{2n+r+1}\frac{z_0}{l_B}\right). \tag{S.22}$$

We now consider and compare the analytical forms of $I_{n,n+r}$ and J_r. When $r = 1$, we obtain the following equation for the the overlap integrals of the adjacent states

$$I_{n,n+1} = \sum_{i=0}^{n} a_i, \tag{S.23}$$

where

$$a_i = \exp\left(-\frac{z_0^2}{4l_B^2}\right)\sum_{i=0}^{n}\frac{(-1)^i}{i!(i+1)!}\left(\frac{z_0}{\sqrt{2}l_B}\right)^{2i+1}\frac{\sqrt{n!(n+1)!}}{(n-i)!}, \tag{S.24}$$

which can be rewritten as

$$a_i = \exp\left(-\frac{z_0^2}{4l_B^2}\right)\frac{(-1)^i}{i!(i+1)!}\left(\frac{z_0}{\sqrt{2}l_B}\right)^{2i+1}\frac{n!}{(n-i)!}(n+1)^{\frac{1}{2}}. \tag{S.25}$$

Correspondingly the Bessel function of order $r = 1$ can be written as the following summation,

$$J_1 = \sum_{n=0}^{\infty} b_i, \tag{S.26}$$

where

$$b_i = \frac{(-1)^i}{i!(i+1)!}\left(\frac{z_0}{\sqrt{2}l_B}\right)^{2i+1}(n+1)^{\frac{2i+1}{2}}. \tag{S.27}$$

We note that Eq. (S.27) is strikingly similar to Eq. (S.25), in particular if $n = 0$, when

$$a_{i=0} = \exp\left(-\frac{z_0^2}{4l_B^2}\right)\left(\frac{z_0}{\sqrt{2}l_B}\right)(n+1)^{\frac{1}{2}} \tag{S.28}$$

and

$$b_{i=0} = \left(\frac{z_0}{\sqrt{2}l_B}\right)(n+1)^{\frac{1}{2}}. \tag{S.29}$$

Therefore the initial values in the two series (S.23) and (S.26) converge when z_0/l_B is small, i.e. if the separation between the states is small, or if the length scale of the SHO state is large.

In order that the two series converge to zero at the same rate, and thus result in the same summation, we require the ratio between successive elements of each summation be equal, i.e.

$$\frac{a_{i+1}}{a_i} = \frac{b_{i+1}}{b_i}. \tag{S.30}$$

We find that

$$\frac{a_{i+1}}{a_i} = \frac{-1}{(i+1)(i+2)}\left(\frac{z_0}{\sqrt{2}l_B}\right)^2(n-i) \tag{S.31}$$

and

$$\frac{b_{i+1}}{b_i} = \frac{-1}{(i+1)(i+2)}\left(\frac{z_0}{\sqrt{2}l_B}\right)^2(n+1). \tag{S.32}$$

It is clear that Eqs. (S.31) and (S.32) have a very similar form, in particular when n is large, showing that the two series converge to zero at the approximately the same rate and will thus have a similar summation. Note that for both $I_{n,n+1}$ and J_1, the series will converge rapidly to zero when z_0/l_B is small, irrespective of the value of n.

6 Dynamic barriers determined by other rings of stochastic webs

In some cases, when C in Eq. (5) of the main text is large, Δ_C is also large and the electron can then continue along the filament to the second ring in the stochastic web. Here, $|\Psi|^2$ and the semi-classical trajectories extend to regions beyond the dynamical barrier rather than being confined to the region defined by the first ring along the x-axis, as shown by Eq. (8) in the main text. However, they do vanish at an x value that corresponds to another zero of the overlap integrals, which determines the radius of the associated stochastic web ring in phase space. For example, the ith zero of the Bessel function and i^{th} stochastic web ring of radius ρ_r^i leads to the wavefunction vanishing at the following x value:

$$\Delta x_r^i = \frac{d l_B^2}{2 r z_0^2}(z_0 \rho_r^i)^2. \tag{S.33}$$

7 Equivalence of I and J when on resonance and $r > 1$

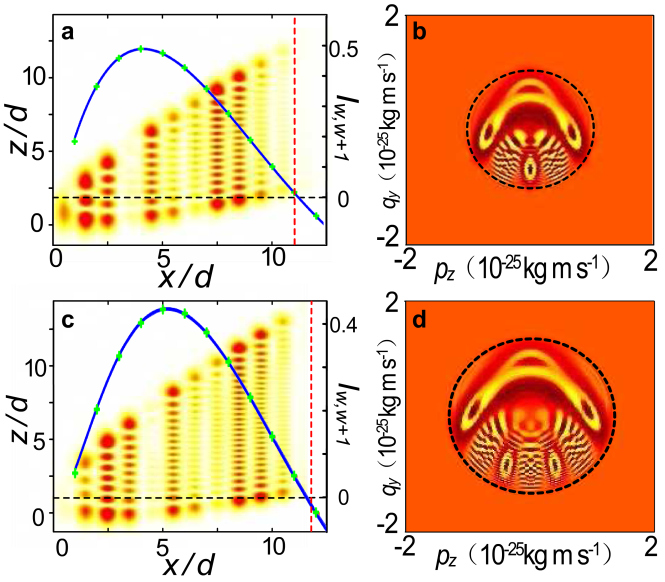

Figure S2. **(a,c)** Colour maps of eigenstate probability density in the $x-z$ plane (red high) calculated for a 14-period SL with $B = 20$ T and $\theta = 30^o$. Green crosses (blue curves) show overlap integrals $I_{w,w+1}$ ($\Xi_{w,w+1}$) referred to right-hand axis. **(b,d)** Colour maps of the corresponding Wigner functions. **(a)** and **(b)** are calculated at the $r = 2$ resonance, and **(c)** and **(d)** at the $r = 3$ resonance. In **(a)** and **(c)**, dashed black lines mark $z = 0$ and dashed red lines mark the orbital widths determined by the dashed stochastic web rings in **(b)** and **(d)**.

In Fig. S2(a), we show $|\Psi|^2$, when $r = 2$ and find that the number of nodes in the z direction increases by two between adjacent wells, meaning that the energy difference is twice the Landau level spacing. The eigenstate vanishes at the 11th quantum well, exactly the position (marked by dashed red line in Fig. S2(a)) where the Bessel function $J_2[\sqrt{2n+3}z_0/l_B]$, and the overlap integral $I_{n,n+2}$ (shown by the green crosses and blue curve in Fig. S2(a)) are zero. This position corresponds to the first stochastic web ring marked by the dashed circle on the Wigner function in Fig. S2(b). Similarly for $r = 3$, the eigenstate vanishes at the 12th quantum well [Fig. S2(c)], corresponding to the first root of $J_3[\sqrt{2n+4}z_0/l_B]$ and the first stochastic web ring shown dashed in Fig. S2(d).

The width of the dynamical barrier is $\propto 1/\tan^2\theta$. However, for high energy states, the de Broglie wavelength for the electron motion can become much shorter than the SL period, which means that the semi-classical model is invalid and the dynamical barrier no longer exists.

For a wide range of parameter space, we find good agreement between $I_{n,n+2}$ and $\Xi_{n,n+2}$, as shown in Fig. S3.

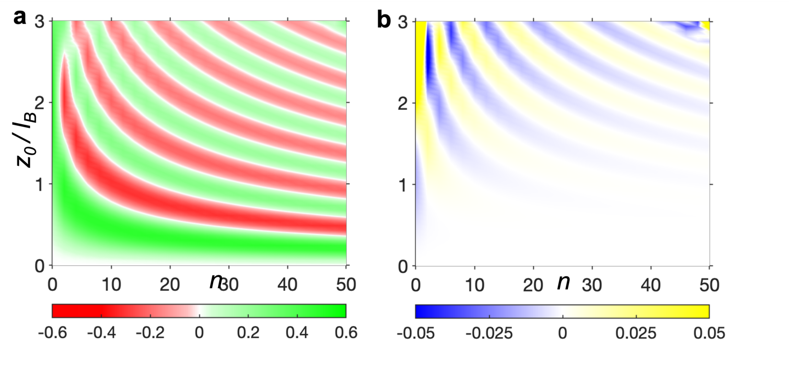

Figure S3. Colour maps (scales below) of **(a)** the overlap integrals $I_{n,n+2}(n, z_0/l_B)$, **(b)** $I_{n,n+2} - \Xi_{n,n+2}$ calculated versus n and z_0/l_B.

References

1. Hardwick, D. P. A. Quantum and semiclassical calculations of electron transport through a stochastic system. Ph.D. thesis, The University of Nottingham (2007).
2. Wannier, G. H. The structure of electronic excitation levels in insulating crystals. *Phys. Rev.* **52**, 3, (1937).
3. Kohn, W. Analytic properties of Bloch waves and Wannier functions. *Phys. Rev.* **115**, 4, (1959).
4. Smith, W. L. Approximate formulae for the overlap integral of two harmonic oscillator wave functions. *J. Mol. Spectrosc.* **225**, 1, (2004).
5. Iachello, F., & Ibrahim, M. Analytic and algebraic evaluation of Franck Condon overlap integrals. *J. Phys. Chem.* **102**, 47, (1998).
